# Industrial and Ruminant Trans-Fatty Acids-Enriched Diets Differentially Modulate the Microbiome and Fecal Metabolites in C57BL/6 Mice

**DOI:** 10.3390/nu15061433

**Published:** 2023-03-16

**Authors:** Farzad Mohammadi, Miranda Green, Emma Tolsdorf, Karine Greffard, Mickael Leclercq, Jean-François Bilodeau, Arnaud Droit, Jane Foster, Nicolas Bertrand, Iwona Rudkowska

**Affiliations:** 1 Endocrinology and Nephrology Unit, CHU de Québec-Université Laval Research Center, Québec, QC G1V 4G2, Canada; farzad.mohammadi.1@ulaval.ca (F.M.);; 2 Département de Kinésiologie, Université Laval, Québec, QC G1V 0A6, Canada; 3 Department of Psychiatry and Behavioral Neurosciences, McMaster University, Hamilton, ON L8S 4L8, Canada; 4 Département de Médecine, Université Laval, Québec, QC G1V 0A6, Canada; 5 Département de Médecine Moléculaire, Université Laval, Québec, QC G1V 0A6, Canada; 6 Faculté de Pharmacie, Université Laval, Québec, QC G1V 0A6, Canada

**Keywords:** industrial trans-fatty acid (I-*t*FA), metabolites, microbiomes, ruminant trans-fatty acid (R-*t*FA), short-chain fatty acids (SCFAs)

## Abstract

Industrially originated trans-fatty acids (I-*t*FAs), such as elaidic acid (EA), and ruminant trans-fatty acids (R-*t*FAs), such as *trans*-palmitoleic acid (TPA), may have opposite effects on metabolic health. The objective was to compare the effects of consuming 2–3% I-*t*FA or R-*t*FA on the gut microbiome and fecal metabolite profile in mice after 7 and 28 days. Forty C57BL/6 mice were assigned to one of the four prepared formulations: lecithin nanovesicles, lecithin nanovesicles with EA or TPA, or water. Fecal samples and animals’ weights were collected on days 0, 7, and 28. Fecal samples were used to determine gut microbiome profiles by 16S rRNA sequencing and metabolite concentrations by GC/MS. At 28 days, TPA intake decreased the abundance of *Staphylococcus* sp55 but increased *Staphylococcus* sp119. EA intake also increased the abundance of *Staphylococcus* sp119 but decreased *Ruminococcaceae* UCG-014, *Lachnospiraceae*, and *Clostridium sensu stricto* 1 at 28 days. Fecal short-chain fatty acids were increased after TPA while decreased after EA after 7 and 28 days. This study shows that TPA and EA modify the abundance of specific microbial taxa and fecal metabolite profiles in distinct ways.

## 1. Introduction

Trans-fatty acids (*t*FAs) are known for their adverse physiological effects [1]. Nevertheless, not all *t*FAs have the same effects on metabolism [2]. Specifically, *t*FAs include industrial *t*FAs (I-*t*FAs) that are made by partial hydrogenation of oil in the industry, such as elaidic acid (EA; *trans*-18:1n-9) [3], and ruminant *t*FAs (R-*t*FAs) produced by bacterial hydrogenation of unsaturated fatty acids, such as *trans*-palmitoleic acid (TPA, *trans-*16:1n-7) and *trans*-vaccenic acid (TVA, *trans-*18:1n-7) [3]. The global consumption of *t*FA is reported to represent 1.4% of the total energy intake (0.2–6.5% range) [4] and is mostly I-*t*FAs. Indeed, consumption of R-*t*FAs is estimated at <0.5% of the total energy intake [5]. Contrary to I-*t*FAs, recent observational studies showed that circulating TPA may lower insulin resistance, atherogenic dyslipidemia, and the incidence of type 2 diabetes [6]. Nevertheless, the differences in the mechanisms of action between I-*t*FA and R-*t*FA are unknown. Modifications in the gut microbiome (GM) may be potential novel mechanisms that may modulate the effects of I-*t*FA and R-*t*FA on metabolic pathways.

Indeed, quantitative and qualitative compositions of GM have been shown to be related to insulin resistance [7], inflammatory [8], and metabolic syndrome pathways [9]. Microbiota–host interactions influence nutrient uptake and impact host metabolism. Dietary nutrients undergo microbial fermentation, resulting in the production of metabolites, such as short-chain fatty acids (SCFAs). For example, butyrate, one of the major SCFAs, is known for its various physiological functions: serving as fuel for colonic epithelial cells, inducing the proliferation of intestinal cells, and acting as an anti-inflammatory immune modulator [10]. Moreover, bacterial fermentation of branched amino acids, such as valine, leucine, and isoleucine, results in branched SCFAs. Thus, identifying key bacterial taxa that respond to nutrients is important for understanding the impact of dietary factors on host health. In addition, investigation of fecal metabolomics provides functional readouts of the metabolic interplay between the host, diet, and the GM and may complement sequencing-based approaches. Notably, fecal metabolites have been used as biomarkers of metabolic disorders, such as type 2 diabetes and inflammation, in animal and human studies [11,12].

Previously, the respective effects of I-*t*FA and R-*t*FA on changes in GM and fecal metabolites have been investigated. First, the intake of EA (I-*t*FA, diet containing low (4%) or high (23%) levels of partially hydrogenated soybean oil) was shown to increase the abundance of harmful bacteria such as *Proteobacteria* in C57BL/6 mice for 8 weeks [1]. The same study demonstrated that EA also numerically decreased fecal butyric and valeric acids [1]. In another study, results show that the consumption of a diet rich in *Decaisnea insignis* seed oil that composed of 55% palmitoleic acid (16:1n-7), 12% palmitic acid (C16:0), and 29% oleic acid (C18:1, n = 9) of total fat (3–12 g/kg) increased the abundance of beneficial bacteria such as *Lactobacillus* after 12 weeks in Kunming male mice [13]. Yet, to our knowledge, there is no study that directly compares the effects of I-*t*FA and R-*t*FA on GM and fecal metabolites profiles to examine the potential mechanisms of action.

Thus, it is hypothesized that the intake of I-*t*FA and R-*t*FA will differentially modify GM and fecal metabolites profiles. Specifically, the main objective of the study is to examine differences in GM and fecal metabolites profiles after intakes of 2–3% of the total energy of EA (I-*t*FA) or TPA (R-*t*FA) for 7 and 28 days in C57BL/6 mice. Yet, it is well known that assessment of oral intake of a solid diet needs individual housing of animals, as well as frequent weighing of mangers which makes it difficult to use solid diet [14]. Therefore, delivering fatty acids in the form of solutions has been suggested as an alternative method [15]; however, this strategy does not address the poor solubility of fatty acids in the watery environment of the gastrointestinal tract. Consequently, nanovesicles prepared by hydration of lipid films followed by extrusion through polycarbonate membranes have been proposed to overcome this challenge. Here, we implement these lipid nanovesicles to understand the full impact of lipid delivery on GM and metabolite factors and assess their effectiveness as a nutrient vehicle in vivo.

## 2. Materials and Methods

### 2.1. Materials

EA and TPA, which are free fatty acids with a purity of over 99%, were obtained from Nu-check Prep, Inc. (Elysian, MN, USA) under product numbers U-47-A, U-49-A, and U-41-A, respectively. Soy lecithin Ultralec^®^ F (97% purity, CAS# 8030-76-0, product no: 2516, Medisca, Montréal, QC, Canada) was generously donated by Medisca, Inc. The commercial Teklad Global 18% Protein Rodent Diet, which was adjusted for calories, with 58% of calories from carbohydrates, 24% from proteins, and 18% from fats, was purchased from Envigo International Holdings, Inc. (Madison, WI, USA). Further details about the diet can be found online (www.envigo.com (accessed on 11 March 2023)). All remaining reagents and solvents were procured from Sigma-Aldrich (St. Louis, MO, USA) or Fisher Scientific (Waltham, MA, USA).

### 2.2. tFA Preparation

Lipid film technique is adapted from common methodologies used to fabricate liposomes [16]. Briefly, soy lecithin and *t*FA (EA and TPA) were added to chloroform/methanol (8:2 *v*/*v*) or methanol to reach a concentration of 40 mg/mL in a round-bottom flask. A mixture of lecithin and *t*FA solutions for each single fatty acid at a ratio of 86:14 (*w*/*w*) was prepared [17]. The solvent was removed on a rotary evaporator, and the lipid film was hydrated with distilled water. The vesicles were extruded on 400, 200, and 100 nm polycarbonate filters, using a Liposofast-50 high-pressure extruder (Avestin, ON, Canada). The composition of fatty acid in vesicle formulations was determined by gas chromatography as previously described by Chotard et al. [17].

### 2.3. Animals and Diets

All animal experiments in this study were carried out in accordance with the guidelines of the Canadian Council on Animal Care and approved by Université Laval (Protocol #17-019-3). Seven-week-old healthy male mice were purchased from Charles River (Saint-Constant, QC, Canada) and housed in a facility with a controlled environment (22 °C, 12 h day/night cycle) with ad libitum access to food and water. Forty C57BL/6 mice were divided into four equal groups. Each group was given a normal diet (Teklad Global 18% Protein Rodent Diet, Envigo, Madison, WI, USA) with water, blank nanovesicles (only lecithin) as a negative control group or different formulations of *t*FA: containing 14 wt% TPA or EA. Animals were followed for 28 days. All vesicle formulations had a phospholipid concentration of 1 wt%. The diet was standardized across all groups, and the only variation was in the solutions provided for the animals to consume. Animal weight was measured every 3 days and fecal samples were collected at days 0, 7, and 28 for further analysis on short- and long-term changes in microbiota and metabolites [18,19,20].

### 2.4. 16S rRNA Sequencing and Amplicon Sequence Variant (ASV) Processing

Bacterial DNA was isolated from cecal and fecal samples utilizing methods that have been previously reported, albeit with some modifications [21]. Briefly, the samples were initially transferred into screw cap tubes that contained 2.8 mm ceramic beads, 0.1 mm glass beads, GES, and sodium phosphate buffer, in agreement with the reported procedures. Following this, the samples were subjected to bead beating and centrifugation, with the supernatant subsequently subjected to further processing through utilization of the MagMAX Express 96-Deep Well Magnetic Particle Processor (Applied Biosystems, Waltham, MA, USA) along with the Multi-Sample kit (Life Technologies #4413022, Carlsbad, CA, USA)). Amplification of the 16S rRNA gene sequences was performed in accordance with published protocols, incorporating modifications that were described by Whelan et al. [22,23]. For this process, 50 ng of DNA was used as a template with 1U of Taq polymerase (Thermofisher, Waltham, MA, USA), 1x buffer, 1.5 mM MgCl2, 0.4 mg/mL BSA, 0.2 mM dNTPs, and 5 pmol each of 341F and 806R Illumina adapted primers. The reaction was carried out with an initial step at 94 °C for 5 min, followed by five cycles of 94 °C for 30 s, 47 °C for 30 s, and 72 °C for 40 s. Another 25 cycles were executed at 94 °C for 30 s, 50 °C for 30 s, and 72 °C for 40 s, with a final extension of 72 °C for 10 min. The resulting PCR products were visualized on a 1.5% agarose gel to verify amplicon size. Positive amplicons were normalized using the SequalPrep normalization kit (ThermoFisher #A1051001, Waltham, MA, USA) and sequenced on the Illumina MiSeq platform at the McMaster Genomics Facility (Hamilton, ON, Canada).

The raw FASTQ files from Illumina were subsequently processed through DADA2, a Bioconductor package [24,25]. The sequences underwent truncation, removal of PCR primers, and discarding of expected errors. The estimation of the error rates of the sequences was accomplished using the learnErrors function. The filtered sequences were then dereplicated using the *derepFastq* function, and unique sequences were generated. The generation of amplicon sequence variants (ASVs) from the sequences was accomplished using the dada function. The forward and reverse reads were independently processed. Using the *mergePairs* function, reverse and forward reads were merged to refine ASVs, in which a count table was then generated. Chimeric sequences were removed using the *removeBimeraDenovo* function [24,25]. Taxonomic assignments up to the genus level were made using the Ribosomal Database Project (RDP) classifier and Silvia reference database (version 1.3.8). To prepare for further analysis, the ASV table was generated by removing singleton ASVs with the use of the phyloseq package in R.

### 2.5. Metabolites Analysis

#### 2.5.1. Analysis of SCFA

Samples were prepared in a 20 mL headspace screw cap bottle containing 400 mg of sodium chloride, 71 µL of phosphoric acid 85%, and 910 µL of water. A volume of 19 µL of an internal standard, 2-ethylbutyric acid (1.11 mg/mL) purchased from Fisher Scientific (Waltham, MA, USA) was added to the sample. Finally, 5 mg of freeze-dried feces sample was added and shacked at low speed for 5 min. Volatile Free Acid Mix from Sigma (Oakville, ON, Canada) containing acetic, propionic, butyric, isobutyric, isovaleric, valeric, isocaproic, caproic, and heptanoic acids was used to build a calibration curve. The standard solutions were prepared similarly to samples by counterbalancing the amount of water added to reach a total volume of 1 mL. Sample analysis by headspace injection was carried out with Pal 3 injection system and performed on an Agilent gas chromatograph (GC) 7890B coupled with a single quadrupole mass spectrometer 5977B (Santa Clara, CA, USA). Headspace stability was reached by a 40-min incubation at 85 °C under agitation. A sample volume of 1 mL was introduced with a split ratio (3:1) into the injector set at 250 °C. The carrier gas (helium) flow rate was fixed at 1.75 mL/min. Chromatographic separation was accomplished with an HP-Innowax column (30 m × 0.25 mm × 0.25 m) from Agilent with the following oven program. The initial temperature of 50 °C was held for 8 min and increased to 260 °C at a rate of 30 °C/min. The oven remained at this final temperature for 5 min. Mass spectra were recorded in scan mode (29–200 amu) with an electron impact ion source set at 70 eV.

#### 2.5.2. Analysis of Other Metabolites

Analysis of other metabolites was carried out as previously described (19) with few modifications for feces. Briefly, 1 mg of freeze-dried feces was mixed with 20 µL of a xylose solution in water and 20 µL of myristic-d27 and δ-tocopherol solution in ethanol as internal standards, each compound at a concentration of 0.05 mg/mL. To prevent oxidation, 15 µL of butyl-hydroxy-toluene (1% *w*/*v* in ethanol) was also added. A first extraction was performed by adding cold solvents (−20 °C): 150 µL of methanol and 55 µL of ethanol. The sample was subjected to vortexing for 5 min at 4 °C, followed by centrifugation for 2 min at 16,000 rcf. The supernatant was transferred to a new tube and labeled as ‘MeOH/EtOH/H2O extract’. The resulting pellets were rinsed with 150 µL of double distilled water (mean resistivity 18.2 MΩ, Milli-Q, Millipore, Etobicoke, ON, Canada) and vortexed and centrifuged at room temperature at 16,000 rcf. The aqueous supernatant was added to the MeOH/EtOH/H2O extract and stored at 4 °C. A last extraction with 50 µL of isopropanol was carried out on the pellet, after the vertexing and centrifuging at 16,000 rcf steps; the resulting supernatant was transferred to a new tube, labelled as ‘IPA extract’ and dried. Once the alcohol evaporated, the MeOH/EtOH/H2O extract was added to the evaporated IPA extract and vortexed. A final centrifugation was carried out and the resulting supernatant was transferred to a new tube. A volume of 116 µL of the total extract was collected, corresponding to about 0.3 mg of feces of each sample was added to a new tube and the rest of the sample was used to constitute a pool for a spiked calibration curve. The two-stage derivatization process was executed in accordance with the extensive method outlined by Fiehn (Agilent Technologies, Inc., Santa Clara, CA, USA, Manual Part Number: G1676-90001, 2013). Chromatography was conducted employing an Agilent 7890B GC oven that was coupled to an MS 5977B Mass Selective Detector (Agilent Technologies, Santa Clara, CA, USA) following the manufacturer’s instructions (Agilent Technologies, Inc., Manual Part Number: G1676-90001, 2013). Metabolites were detected utilizing the AMDIS software (version 2.71) and characterized based on their mass spectra patterns and retention index matches in the Agilent G1676AA Fiehn GC/MS Metabolomics RTL Library (Agilent Technologies, Santa Clara, CA, USA) and the NIST/EPA/NIH Mass Spectral Library (Version 2.2, 2014) (National Institute of Standards and Technology (NIST), Gaithersburg, MD, USA). To determine quantitative values, standards of the same compound were utilized for quantification. Semiquantitative values were expressed as the ratio of the compound response (area under the curve or peak height) to that of the corresponding internal standard (compounds with the same spectral and retention time). The sole distinction between quantitative and semiquantitative approaches is that no calibration curve slope is employed in the latter. The sample contained 10 bile acids named by number considering similar defragmentation spectrums and retention times too close to allow a differential identification using the library.

### 2.6. Statistical Analyses

All statistical analyses for weight change were performed with SPSS software (version 18, SPSS Inc., Chicago, IL, USA), and Shapiro–Wilk test was performed to evaluate the normality of distribution before performing statistical analyses. Continuous variables were considered for normality, and nonnormal variables were log-10 transformed for significance testing. ANOVA followed by Bonferroni post hoc test was performed to compare animal weight at days 0, 7, and 28 between each group: lecithin, EA, TPA, and water.

All microbiome analysis analyses were completed within R version 3.4.3. Alpha and beta diversity were calculated using the raw ASV data using the vegan package. Alpha diversity metrics included the Inverse Simpson index and the Shannon index. Differences between strains were assessed using a Skillings–Mack test to account for incomplete block design with a significance cut-off of *p* < 0.05. Pairwise differences were also assessed using a Wilcoxon post-hoc test. Beta diversity between samples was explored using principal coordinate analysis (PCoA) with Jaccard, Bray–Curtis, and Aitchison distance metrics applied to ASV count data. To assess the influence of diet on beta diversity across the supplementation period, a blocked permutational multivariate analysis of variance (PERMANOVA) was conducted using the distance metrics function *adonis()* from the phyloseq package, stratified such that permutations were restricted to age-matched supplementation groups. Pairwise comparisons were implemented to assess drivers of omnibus group differences significant to *p* < 0.05.

Prior to differential abundance analysis, ASV tables were further preprocessed using the retain resolve method, as previously described [26]. In brief, taxa were sequentially filtered and glommed to obtain a final dataset that retains biologically significant taxonomic classifications while removing noise in the form of spurious sequences and highly partitioned subgroups. Differential abundance analysis was executed using a consensus technique, combining models constructed using Linear models for Differential Abundance analysis (LinDA) [27] and a modified limma/voom pipeline [28]. All taxa were assessed for significant changes between each dietary group and the control group (H_2_O) across sampled timepoints. This was achieved by setting the H_2_O group as a baseline and testing the marginal effect of each diet (i.e., the interaction between diet and timepoint condition) at each sampling day. To account for compositionality, data were either centered log-ratio or log-ratio transformed prior to mean comparisons. Repeated measures in the LinDA and limma/voom models were accounted for by either integrating a random effect term (animal ID) into the model formula or by using the *duplicateCorrelation()* function, forcing the magnitude of the random effect to be the same across all taxa, respectively. Criteria for significantly different taxa between groups were set at a Benjamini–Hochberg (BH) corrected *p*-value < 0.05 and an effect size analog (log-2-fold change or change coefficient) of >1. A final list of consensus taxa was then constructed using the mutually significant taxa between models at each timepoint.

Metabolites analyses were performed by the Metaboanalyst platform (online: www.metaboanalyst.ca (accessed on 17 January 2022)). The difference in metabolites level at baseline with day 7 and day 28 was evaluated by paired t-test for each group. *p*-values ≤ 0.05 were considered as statistically significant.

### 2.7. Machine Learning Analyses

Supervised machine learning models were performed using BioDiscML to identify optimal relevant predictive signatures of microbiomes and metabolites. The tool reports various models and signatures with many metrics obtained through multiple evaluation methods (e.g., 10-fold cross-validation, leave-one-out cross-validation, bootstrapping). Different scenarios were conducted to find the optimal model. The scenarios were microbiome data at days 0 and 7; microbiome data at days 0, 7, and 28; metabolite data at days 0 and 7; and metabolite data at days 0, 7, and 28.

## 3. Results

### 3.1. Fatty Acid Composition of Vesicles Is Stable

**Figure 1:**
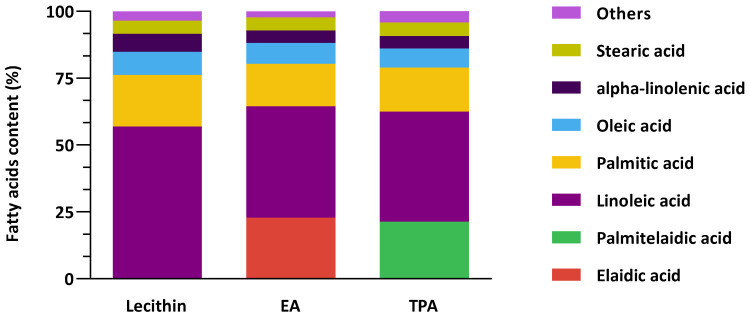
The fatty acids composition of control vesicle (Lecithin) and elaidic acid (EA) or *trans*-palmitoleic acid (TPA)-enriched vesicles.

Figure 1 shows the combined fatty acid composition of lecithin vesicles and the encapsulated *t*FA. Control vesicles are mainly composed of linoleic acid, palmitic acid, oleic acid, EA, TPA, alpha-linolenic acid, and stearic acid. *t*FAs encapsulated are comparable between formulations since encapsulated EA and TPA were 22.9% and 21.4%, respectively. Overall, these results demonstrate that the composition of fatty acids in the vesicles was comparable except for EA and TPA in each corresponding vesicle.

### 3.2. Animal Weight Is Similar in All Groups

**Figure 2:**
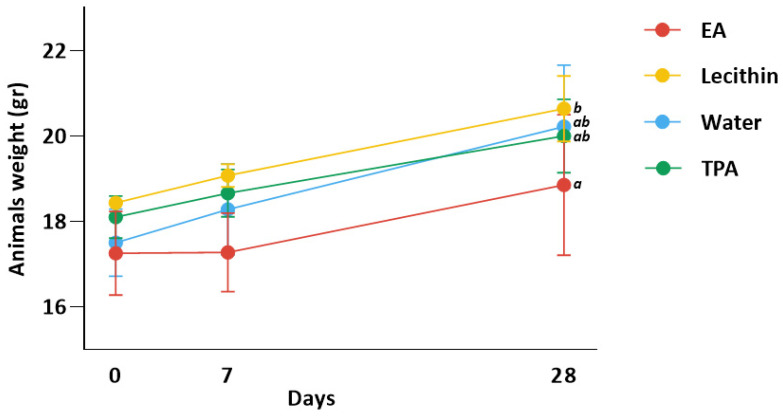
Change in animal weight after 7 and 28 days. Data represent means ± SD. ^ab^ Means at day 28 with different subscript letters are significantly different between groups. Specifically, the ANOVA test revealed a significant difference between the EA and lecithin groups at day 28 (*p*-value < 0.05). Yet, there was no significant difference in mean weight between the EA and TPA groups.

Figure 2 shows animal weight for all groups. Animals had an initial (day 0) weight of 18.2, 17.2, 18.3, and 17.8 g for lecithin, EA, TPA, and water group, respectively. Animals gained 0.64, 0.02, 0.56, and 0.78 g after 7 days and 2.20, 1.60, 1.90, and 2.72 g after 28 days in lecithin, EA, TPA, and water groups, respectively. ANOVA test indicated a significant difference between EA and lecithin groups at day 28 (*p*-value < 0.05). Moreover, there was no significant difference between EA and TPA groups in weight.

### 3.3. tFA Intake Does Not Impact Alpha Diversity but Modifies Beta Diversity

**Figure 3:**
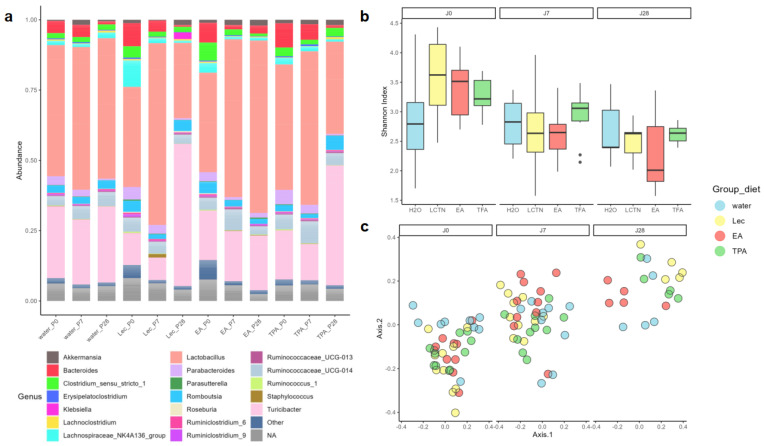
Compositional differences in GM between mice supplemented with water, lecithin vesicle, TPA, and EA: (**a**) compositional bar chart; (**b**) alpha diversity, Shannon index; and (**c**) beta diversity, Bray–Curtis.

Compositional differences in GM between mice supplemented with water, lecithin vesicle, TPA, and EA were investigated by 16S rRNA gene sequencing. There were no differences in alpha diversity between supplement groups (Skillings–Mack (SM) test, *p* = 0.2695), although a decrease in alpha diversity was observed in an omnibus comparison between timepoints (SM, *p* < 0.01). The further pairwise comparison revealed that this result was driven by differences in the lecithin, TPA, and EA groups from day 0 to day 7 and day 0 to day 28 (Wilcoxon rank-sum test with *p*-value correction, *p* < 0.01), though no differences were observed in animals supplemented with water (*p* > 0.1). This is supported by visualization of the data in Figure 3a, wherein there is a notable downwards trend in alpha diversity in all intake groups, including the blank lecithin, while in the water group alpha diversity remains relatively constant over time.

Despite a lack of discernable clustering of samples by diet in Bray–Curtis ordination plots (Figure 3b), there was an effect of diet group on beta diversity (*p* < 0.05, blocked PERMANOVA). However, Figure 3b also presents a notable shift in microbiome composition across sampling timepoints, which was also significant (*p* < 0.001, blocked PERMANOVA). Omnibus differences in diet were driven by only a few group-wise differences, including water vs. lecithin and lecithin vs. TPA at day 7 (*p* < 0.05) as well as water vs. lecithin and lecithin vs. EA at day 28 (*p* < 0.05). Pairwise comparisons also revealed that longitudinal shifts in composition were present in groups supplemented with EA (*p* < 0.05, day 0 to day 7 and day 0 to day 28), lecithin, and TPA (both *p* < 0.05 from day 0 to day 7, day 0 to day 28, and day 7 to day 28), but not in the water-supplemented group. Together, these findings suggest that although subtle shifts in the GM may occur with *t*FA intake, these changes are not significant at the levels of overall intra- and inter-sample microbiome diversity and are not substantially different from changes introduced by supplementing with blank lecithin. 

### 3.4. tFA Intake Impacts Abundance of Select ASVs

**Figure 4:**
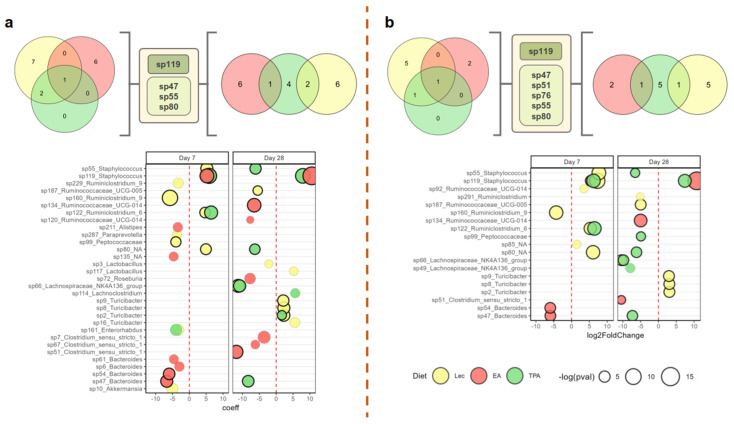
Differential abundance (DA) analysis of differences in amplicon sequence variant (ASV)-level microbial abundance between supplement groups and water across sampling timepoints. Significant differentially expressed taxa for models constructed using LinDA (**a**) and limma/voom (**b**). Venn diagrams (top) show mutually significant taxa between timepoints (day 7, left and day 28, right) and diets. Bubble blots (bottom) display change coefficients/log-fold change of differentially abundant taxa. Bubble size represents the negative logarithm of *p*-values for each taxon. Bolded bubbles represent taxa that were found to be mutually significant between models.

To explore more fine-grained impacts of *t*FA intake on the microbiome, we conducted a differential abundance (DA) analysis that compared differences in ASV-level microbial abundance between supplement groups and water across sampling timepoints. The results of this analysis are shown in Figure 4. According to consensus analysis (bolded circles), *t*FA intake impacted the abundance of 9 ASVs at day 7 and 13 ASVs at day 28. However, among these ASVs, only two (*Bacteroides sp47* and *sp54*) were uniquely impacted by either TPA or EA intake compared to the lecithin control at day 7, while only six (including *Ruminococcaceae UCG-014 sp134*, *Bacteroides sp47* and *sp54*, as well as *Clostridium sensu stricto sp51*) met these criteria at day 28. On day 7, all three intake groups displayed a marked increase in *Staphylococcus sp119*, while both TPA and lecithin groups showed an increase in *Ruminiclostridium 6 sp122*. At day 28, *Lachnospiraceae NK4A136 group sp66* displayed a decreased abundance in both the lecithin and TPA groups. Interestingly, some ASVs, such as *Bacteroides sp47*, were only decreased in the EA group at day 7 and only decreased in the TPA group at day 28. Furthermore, despite showing an increase across all three experimental groups at day 7, *Staphylococcus sp119* only persisted at increased levels in *t*FA intake at day 28 but not in the lecithin group. Together, these results show that while global microbiome features were not strongly impacted by *t*FA intake, supplementation does alter the longitudinal community structure of specific microbial taxa that may have implications for overall functional output. (The abundances of all the (consensus) DA taxa across diet groups and timepoints are shown in Figure S4). 

### 3.5. tFA Intake Impacts on Fecal Metabolites

**Figure 5:**
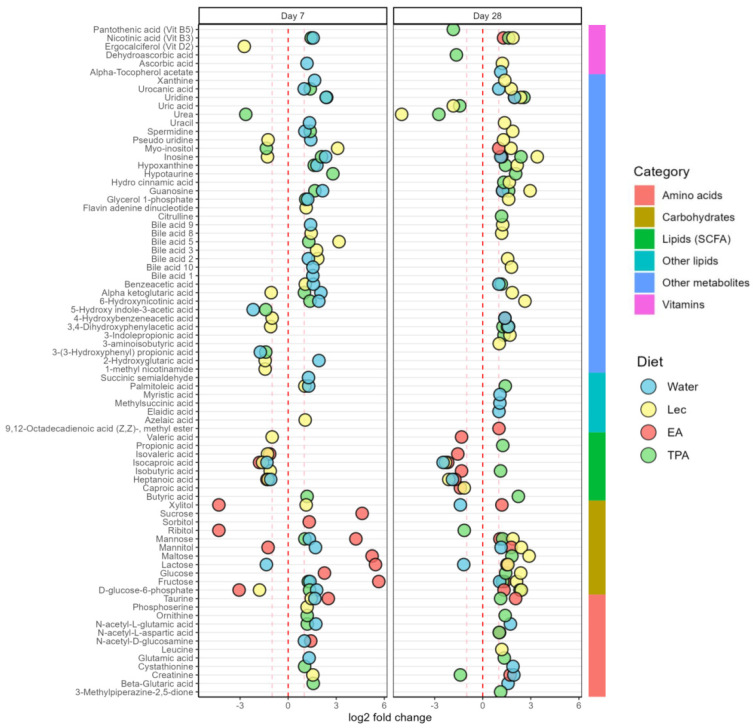
Fold changes for semiquantitative (SQ) lipids, carbohydrates, amino acids, vitamins and derivatives, bile acids, purine compounds, and other organic metabolites after lecithin, EA, TPA, and water intake after 7 and 28 days. Zero shows the direction of change, and lines at 1 and −1 represent a doubling or halving in metabolite abundance. Changes in all values are significant compared to day 0 (*p* < 0.05). (Fold-change values are presented in Tables S1 and S2 and fold-change for quantitative values for is shown Figure S3).

The changes in fecal metabolites after *t*FA intake for 7 and 28 days compared to the baseline are presented in Figure 5.

#### 3.5.1. Lipids and Fatty Acids

After 7 days, TPA intake increased butyric acid. On the other hand, heptanoic acid and isocaproic acid decreased in the water, lecithin, and EA-supplemented groups after 7 days. Furthermore, the lecithin and EA intake also decreased isovaleric acid. Lecithin intake also decreased isobutyric acid and valeric acid on day 7. After 28 days, the TPA intake increased in butyric acid, isobutyric acid, and propionic acid. Oppositely, heptanoic acid and isocaproic acid decreased in the water, lecithin, and EA-supplemented groups after 28 days. Furthermore, lecithin and EA intake decreased caproic acid after 28 days. EA intake also decreased isobutyric acid, isovaleric acid, and valeric acid after 28 days.

Other lipids, such as azelaic acid and palmitoleic acid, were increased with lecithin intake after 7 days. Furthermore, water intake increased palmitoleic acid and succinic semialdehyde after 7 days. After 28 days, water intake increased levels of myristic acid, elaidic, acid and methyl succinic acid. Furthermore, TPA intake increased palmitoleic acid on day 28. Finally, methyl ester was increased following EA intake after 28 days.

#### 3.5.2. Carbohydrates and Derivatives

Fecal glucose levels were increased on day 7 by EA intake; yet on day 28 glucose levels were increased by lecithin and TPA intake. Fructose was increased by water, TPA, and EA intake on days 7 and 28, whereas fructose was increased by lecithin only on day 28. Mannose was increased after 7 days by water intake, after 7 and 28 days by EA and TPA intake, and after 28 days by lecithin intake. Mannitol increased after 7 days by water intake along with 28 days by water and lecithin intake. Even if mannitol decreased after 7 days by EA intake, it increased after 28 days. Sucrose and sorbitol were increased on day 7 after EA intake. Lecithin increased the level of xylitol after 7 days, but water decreased levels of xylitol after 28 days. Furthermore, xylitol was decreased after 7 days by EA intake; yet, after 28 days xylitol was increased. Furthermore, ribitol decreased after 7 days when mice received EA and after 28 days when mice received TPA intake. After 7 days, water and TPA intake increased D-glucose-6-phosphate. Meanwhile, lecithin and EA intake decreased D-glucose-6-phosphate after 7 days. Furthermore, after 28 days, lecithin, TPA, and EA intake increased D-glucose-6-phosphate. Maltose levels increased after 7 days by EA intake as well as after 28 days by lecithin and TPA intake. On the other hand, lactose levels were decreased by water while it was increased by EA intake after 7 and 28 days. Still, lecithin also increased lactose on day 28.

#### 3.5.3. Amino Acids and Derivatives

Beta-glutaric acid was increased by TPA intake on day 7 and by water on day 28. Creatinine was increased on day 7 by lecithin as well as on day 28 by lecithin and water. However, creatinine was decreased by TPA intake on day 28. Cystathionine was increased by TPA intake on day 7, whereas water increased cystathionine on day 28. Glutamic acid was increased by water after 7 days and TPA intake after 28 days. In addition, N-acetyl-D-glucosamine was increased after water and EA intake only on day 7. Furthermore, EA intake increased N-acetyl-L-aspartic acid after 28 days. Water also increased N-acetyl-L-glutamic acid after both 7 and 28 days; yet TPA intake increased N-acetyl-L-glutamic acid on day 7. Furthermore, ornithine was increased after TPA intake on days 7 and 28. Additionally, lecithin increased phosphoserine on day 7. Likewise, taurine was increased by water, lecithin, and EA intake on day 7. Meanwhile, EA and TPA intake increased taurine on 28 days. Citrulline increased after TPA intake on day 28. Furthermore, 3-aminoisobutyric acid was increased by lecithin on day 28. Finally, TPA intake increased 3-methylpiperazine-2,5-dione after 28 days.

#### 3.5.4. Vitamins

Ascorbic acid (vitamin C) was increased after 7 days on the water diet and after 28 days on the lecithin diet. On the other hand, dehydroascorbic acid decreased following TPA intake on day 28. Alpha-tocopherol (vitamin E) was increased on the water diet after 28 days. Furthermore, ergocalciferol (vitamin D2) was decreased after lecithin intake on day 7. Nicotinic acid (vitamin B3) was increased after water intake on day 7, lecithin intake on day 28, EA intake on day 28, as well as TPA intake on days 7 and 28. Finally, pantothenic acid (vitamin B5) was decreased after TPA intake for 28 days.

#### 3.5.5. Bile Acids

The number of bile acids that increased was four, four, and one following water, lecithin, and TPA intake on day 7, respectively. Yet, after 28 days, only four bile acids were increased after the lecithin intake.

#### 3.5.6. Purine Compounds

On day 7, guanosine was increased after water and TPA intake. Further after 28 days, increased guanosine was observed after lecithin, EA, and TPA intake. Xanthine was increased following water on day 7 and lecithin intake on day 28. Hypoxanthine was increased by TPA intake after 7 and 28 days. Similarly, hypoxanthine was also increased by water on day 7 and lecithin on day 28. Inosine was increased by water and TPA intake after 7 and 28 days as well as EA intake on day 28 only. Interestingly, lecithin intake decreased and increased inosine on days 7 and 28, respectively. Uric acid was decreased after lecithin and TPA intake on day 28.

#### 3.5.7. Organic Compounds

Alpha-ketoglutaric acid was increased following water and TPA intake on day 7. Even though lecithin decreased the level of alpha-ketoglutaric acid on day 7; fecal alpha-ketoglutaric acid increased on day 28. Benzene acetic acid was increased after water intake on days 7 and 28. Yet, lecithin on day 7 and TPA intake on day 28 also increased benzene acetic acid.

Flavin adenine dinucleotide increased after lecithin intake on day 7. Furthermore, water and TPA intake increased glycerol 1-phosphate on day 7 and lecithin increased glycerol 1-phosphate on day 28. Hydrocinnamic acid also increased following lecithin and TPA intake on day 28. Hypotaurine was increased by TPA intake after 7 and 28 days. Myo-inositol increased following lecithin intake after days 7 and 28 as well as EA intake after 28 days. On the other hand, TPA intake decreased myo-inositol and urea on day 7. Pseudo-uridine increased water control on day 7. In addition, pseudo-uridine was increased by lecithin after 28 days, even if pseudo-uridine was decreased by lecithin on day 7. Spermidine was increased by water and TPA intake on day 7 as well as lecithin on day 28. Likewise, uracil was increased after water on day 7 and lecithin on day 28. Urea was decreased by TPA intake on day 7 as well as with lecithin and TPA intake on day 28. Uridine was increased after TPA and water intake after 7 and 28 days, whereas EA increased uridine after 28 days only. Urocanic acid was increased after water on days 7 and 28, after TPA intake on day 7, and after lecithin on day 28. On day 7, lecithin decreased 1-methyl nicotinamide as well as 2-hydroxyglutaric acid even though water increased 2-hydroxyglutaric acid. Additionally, 3-indolepropionic acid was increased after lecithin and TPA intake on day 28. Next, 3-(3-hydroxyphenyl) propionic acid and 5-hydroxy indole-3-acetic acid were decreased by water and TPA intake after 7 days. Furthermore, 6-hydroxynicotinic acid was increased by lecithin on day 28. Yet, lecithin also decreased 3,4-dihydroxyphenylacetic acid after 7 days while EA intake and TPA intake increased 3,4-dihydroxyphenylacetic acid on day 28. Similarly, 4-hydroxybenzeneacetic acid was decreased after lecithin intake on day 7 and oppositely EA intake increased 4-hydroxybenzeneacetic acid on day 28. Finally, 6-hydroxynicotinic acid was increased by water and TPA intake on day 7 and by lecithin on day 28 (Figure 5).

### 3.6. Features Identified from Machine Learning Analysis as Markers of TFA Intake

**Figure 6:**
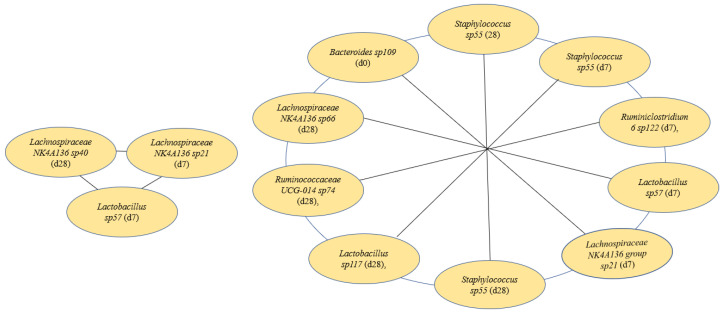
Short (**left**) and long (**right**) signatures detected by machine learning, d = day.

**Figure 7:**
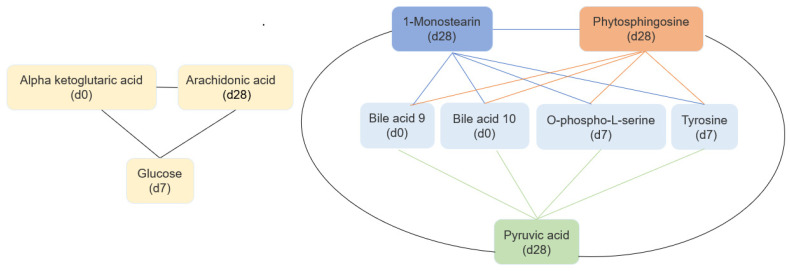
Correlated features between metabolites, similar ranking score (machine learning analyses).

The best machine learning analysis results were obtained with microbiome 0, 7, and 28 days: short signature, average Matthew correlation coefficient (MCC) 0.787 (standard deviation: 0.094). In addition, a long signature was detected with an average MCC of 0.863 (standard deviation: 0.121). The best machine learning analysis results for metabolites 0, 7, and 28 days: average MCC: 0.557 (standard deviation: 0.097) and metabolites 0 and 7 days: average MCC: 0.53 (standard deviation: 0.089). Relevant features from microbiome and metabolites identified from machine learning analyses are presented in Figure 6 and Figure 7.

First, Figure 6 shows a change in microbiomes, where *Lachnospiraceae Lachnospiraceae NK4A136* group sp40 (day 28), *Lachnospiraceae Lachnospiraceae NK4A136 sp21* (day 7), and *Lactobacillaceae Lactobacillus sp57* (day 7) predicted a short signature. Figure 6 also shows *Bacteroidaceae Bacteroides sp109* (day 0), *Staphylococcaceae Staphylococcus sp55* (days 7 and 28), *Ruminococcaceae Ruminiclostridium 6 sp122* (day 7), *Lactobacillaceae Lactobacillus sp57* (day 7), *Lachnospiraceae Lachnospiraceae NK4A136 group sp21* (day 7), *Staphylococcaceae Staphylococcus sp55* (day 28), *Lactobacillaceae Lactobacillus sp117* (day 28), *Ruminococcaceae Ruminococcaceae UCG-014 sp74* (day 28), and *Lachnospiraceae Lachnospiraceae NK4A136 sp66* (day 28) predict a long signature.

Figure 7 shows changes in alpha-ketoglutaric acid (baseline), glucose (day 7), and arachidonic acid (day 28). Moreover, analyses identified another model in which bile acid #10 (baseline), bile acid #9 (baseline), o-phospho-L-serine (day 7), tyrosine (day 7), phytosphingosine (day 28), 1-monostearin (day 28), and pyruvic acid (day 28) were identified as biomarkers. Specific modifications in microbiomes and metabolites profiles after 7 and 28 days is shown in Figures S1 and S2.

## 4. Discussion

Despite the contrasting health effects of I-*t*FA and R-*t*FA, little is known about the mechanisms by which these compounds modulate host metabolism. Consequently, this study explored the impacts of I-*t*FA (EA) and R-*t*FA (TPA) on the fecal microbiome and metabolome, implementing lipid film nanovesicles to facilitate in vivo *t*FA delivery. Our results show that TPA appears to have a more beneficial effect on GM compared to EA, as well as an enhanced production of beneficial fecal metabolites.

Specifically, both TPA and EA increased the abundance of *Staphylococcus sp119* at day 7. However, at 28 days TPA supplementation reduced its relative abundance compared to baseline, whereas abundance was further increased in EA-supplemented animals. TPA also significantly decreased the abundance of *Staphylococcus sp55* at day 28 according to compositional test results. *Staphylococcus* is a major human pathogen and is known for its harmful effects that are related to a variety of diseases [29]. In a study in which mice were fed different types of fat (60% kcal from fat) for 16 weeks, a diet high in coconut fat increased the abundance of *Staphylococcus,* whereas extra-virgin olive oil decreased the abundance of *Staphylococcus* [30]. In another study, a diet high in fat (58%, mainly coconut oil) increased *Staphylococcus* in 16-week-old male offspring [31]. Furthermore, the abundance of *Staphylococcus* was positively correlated with plasma glucose levels in humans [32] and inflammatory cytokines such as TNF-α, IL-1β, and IL-6 in BALB/c mice [33]. Overall, TPA might facilitate relative reductions in *Staphylococcus* with prolonged supplementation compared to EA, which in turn might relate to its anti-inflammatory properties.

In addition, *t*FA supplementation impacted the abundance of multiple ASVs from the *Ruminococcaceae* family, including ASVs classified in the genus *Ruminiclostridium 6* and *Ruminiclostridium 9,* as well as uncultured genus-level groups (UGCs) −014 and −005. However, the impact on populations of UCG-005 and *Ruminiclostridium 9* only reached significance in the lecithin control group, whereas UCG-014 was significantly decreased in the EA group at day 28 and *Ruminiclostridium 6* was significantly increased by TPA supplementation at day 7. In general, *Ruminococcaceae* consists of a group of anaerobic bacteria that exists in the colonic mucosal of healthy individuals and plays an important role in butyrate production [34]. Specifically, *Ruminiclostridium* is generally considered a beneficial bacterium in the gut, involved in the secretion of SCFAs and positive regulation of proper functionality and morphology of intestinal epithelial cells [35]. A previous study with C57BL/6 mice fed a high-fat diet (45 % fat) for 5 months resulted in lower numbers of *Ruminiclostridium 6* [36]. *Ruminiclostridium 6* abundance was also negatively correlated with cholesterol and plasma triglycerides in hamsters fed with a high-fat, high-fructose diet (66% of diet from fat) for 2 weeks [37], suggesting a potentially protective role for this taxon in the context of high-fat feeding. However, *Ruminiclostridium 6* has also been shown to alter energy intake due to the positive correlation to ghrelin levels in rats receiving a high-fat diet (60%, mostly from lard) [38]. Furthermore, the abundance of *Ruminiclostridium 6* was also positively correlated with inflammatory factors such as TNF-α and IL-6 in SPF male BALB/c mice receiving Saikosaponin-d (a major bioactive triterpene saponin) treatment [39]. Thus, the role of *Ruminiclostridium 6* in regulating host lipid metabolism and inflammation may be pleiotropic in nature and context-dependent. It should be noted, however, that the observed increase in the abundance of this genus with TPA supplementation was also observed in lecithin-supplemented controls, thus its mechanistic contribution to the metabolic effects of TPA itself remains unclear.

In contrast, decreased levels of *Ruminococcaceae UCG-014* have been consistently observed in studies exposing rats to high-fat diets over a prolonged period (10–12 weeks) [40,41], and it is a known beneficial bacterium because of its SCFA-producing capabilities [42]. Furthermore, *Ruminococcaceae UCG-014* abundance is also negatively associated with proinflammatory cytokines, such as TNF-α and IL-6 [43]. Thus, the decreased abundance observed only in EA-supplemented animals might contribute to a decrease in SCFA production and potential metabolic perturbation. Considering these results, the diverse impacts of *Ruminococcaceae* genera in response to dietary lipids, either in the presence or absence of caloric surplus, warrant further exploration as a means of modulating host metabolic health.

EA intake also markedly decreased the abundance of *Clostridium sensu stricto sp51. Clostridium sensu stricto* belongs to the order *Clostridiales,* which produces butyrate by fermentation, metabolizing carbohydrates and amino acids [44,45,46,47]. However, *Clostridium sensu stricto 1* has also been characterized as an opportunistic pathogen and has been linked to intestinal inflammation and the development of necrotic enteritis (NE) [48,49]. Additionally, the abundance of *Clostridium sensu stricto* was increased in Wistar rats when fed a high-fat diet (containing 40 kcal%) from soybeans and lard for 8 weeks [50]. These existing data appear to conflict with the suppression of this pathogen in the EA-supplemented group, where one might expect I-*t*FA to promote pathogen outgrowth due to its inflammatory properties. However, this may suggest an interaction between *Clostridium sensu stricto* and populations of other pathogens, such as the aforementioned *Staphylococcus* genus, which shows a marked increase with EA supplementation. Indeed, previous experiments implementing side-by-side challenge models have demonstrated that *Clostridium sensu stricto* is sensitive to the presence of other competing pathogens, and its populations can be suppressed by coinfection with other opportunistic taxa [48]. EA may thus provide a competitive advantage to specific pathogenic strains, subtly disrupting microbiome structure with downstream metabolic effects.

In parallel, SCFAs including caproic, heptanoic, isobutyric, isocaproic, isovaleric, and valeric acids decreased after EA intake. Oppositely, TPA intake increased SCFAs, particularly butyric, isobutyric, and propionic acids. Similarly, a study showed that EA (low (4%) or high levels (23%) of partially hydrogenated soybean oil in diet) decreased fecal butyric acid and valeric acid [1]. SCFAs act as signaling molecules on both the gut cells and other tissue cells [51]. Butyrate is an essential fuel source for colonocytes, whereas propionate is mainly taken up by the liver and utilized as a substrate for lipogenesis as well as gluconeogenesis [52]. Beneficial effects of higher SCFAs have been shown on different pathways of host physiology, such as improved weight loss, glycemic control, and improved metabolism [53,54,55]. Overall, the increase in the production of SCFAs might be due to a higher availability of TPA for gut bacteria since GM did not change the abundance of species of bacteria known to influence SCFA production; yet more studies are needed.

After 28 days of exposure to lecithin and TPA, an increase in IPA was observed. The IPA is a metabolite derived from the tryptophan metabolism by the gut bacteria [56]. This indole derivative has been shown to decrease gut inflammation, prevent gut barrier dysfunction through aryl hydrocarbon receptor (AhR) activation, and modulate the secretion of glucagon-like peptide-1 (GLP-1) [57]. Therefore, IPA production may be involved in modulating host inflammation and thus contributes to the metabolic effects of *t*FAs.

Results also demonstrate changes in the concentrations of sugar acids and polyols in fecal content in both EA- and TPA-treated groups. Specifically, after 7 days of EA intake, polyols including mannitol, xylitol, ribitol, and sorbitol were decreased which may be due to an increase in the abundance of *Clostridia* [58]. Similar to GM results changes in sugar acids and polyols concentrations did not persist after 28 days. For TPA intake, only ribitol was decreased after 28 days; yet other sugars were increased. Ribitol is a part of the chemical structure of riboflavin (vitamin B2) which contributes to energy metabolism [59]. Furthermore, myo-inositol levels in feces were increased after EA. Previous work has shown that the depletion of intracellular myo-inositol levels has been associated with glucose homeostasis, insulin resistance, and diabetes complications [60]. Therefore, dietary EA and TPA exposure may modify energy metabolism pathways.

Creatinine was increased in EA but decreased after TPA intake. Fecal creatinine excretion is elevated when there is a breakdown product of creatine phosphate from muscle and protein metabolism. The results show that TPA intake also decreased urea and increased citrulline in feces short term. Urea is a byproduct from purines and amino acid degradation and usually produces free ammonia when degraded by bacterial urease [61]. Citrulline, a nonprotein amino acid, is involved in the urea cycle as well as arginine and nitric oxide metabolism. TPA also decreased fecal uric acid and increased hypoxanthine. High blood concentrations of uric acid are associated with diabetes and the formation of ammonium acid urate kidney stones [62]. Hypoxanthine is an important substrate for colon barrier function, mucosal repair ability, and a healthy microbiota [63]. In addition, fecal glutamic acid was increased after 28 days of TPA intake. The higher level of blood glutamic acid is associated with a higher Homeostatic Model Assessment for Insulin Resistance (HOMA-IR), indicating lower insulin sensitivity [64]. An increase in fecal ornithine was observed in the TPA group. One study reported that administration of ornithine to mice resulted in mucin secretion, cell proliferation, and goblet cell production, which overall improved intestinal health [65]. Fecal hypotaurine was also increased in the TPA group. Hypotaurine is a transition product in the taurine biosynthetic pathway and has an antioxidant activity [66]. Overall, the metabolism of TPA differs from the EA-treated group; however, whether the concentrations in fecal metabolites corresponded to the changes in the levels in plasma metabolites and their impact on health remain to be investigated.

Results from ML analysis indicate that specific features of the GM, such as the abundance of *Lachnospiraceae NK4A136 group sp21*, *Lactobacillus sp57*, and *Bacteroides sp109*, may predict the various *t*FA intake. *Lachnospiraceae* are known for their beneficial effects, particularly through the production of butyrate [67]. In one study, a diet high (23.60%) in I-*t*FA from partially hydrogenated soybean oil resulted in a decrease in *Lachnospiraceae* after 8 weeks [1]. Similarly, in another study in which mice received 60% of energy from fat (mainly lard), a lower amount of *Lachnospiraceae* was observed after 9 weeks [68]. *Lachnospiraceae* appears to be positively associated with unsaturated fat intake and negatively associated with triglycerides and I-*t*FA exposure [69,70]. *Lactobacillus* is a group of probiotic bacteria known for their beneficial effects by improving the bioavailability of nutrients [71]. Studies have previously shown that supplementation with omega-3 polyunsaturated fatty acids (PUFAs) in the form of fish oil for 15 days increased the amount of *Lactobacillus* [72]. In contrast, in a study in which cells were cultured with EA at concentrations of 100, 200, and 500 mg/L for 24 h, *Lactobacillus* decreased [73]. Interestingly, the combination of EA and vaccenic acids (a form of R-*t*FA) was able to improve the growth of *Lactobacillus* [74]. These results align with the expansion of putative opportunistic pathogens in the EA-supplemented group of the present experiment, which have been shown to concurrently decrement *Lactobacillus* populations [48].

Similarly, the features of the fecal metabolites may also help to distinguish the effects of different *t*FAs. Numerous bile acids, which are natural detergents mainly involved in facilitating the absorption of dietary fat, were identified features in the ML signature. Other metabolites in this signature included compounds involved in the metabolism of energy, carbohydrates, proteins, and fats. Arachidonic acid (fatty acids) may also contribute to inflammation by producing mediators [75,76]. Finally, 1-monostearin (also known as 1-stearoyl-rac-glycerol) is formed as a byproduct in the breakdown of fats. Thus, the modification in these fecal metabolites may be related to biomarkers of *t*FA intake or to changes in metabolism after *t*FA intake. Further studies should examine if these features may predict the intake of *t*FA, either EA or TPA.

Potential limitations and strengths of the study should be considered. First, a cage-to-cage difference might impact the composition since the GM composition is highly sensitive [77]. However, the other potential environmental factors that affect GM composition such as diets, water, light, temperature, bedding, humidity, cage-changing frequency, and animal handlers were controlled for all groups. Another significant limitation of the study is that the vesicles were prepared using lipid films obtained by the evaporation of organic solvents. With this synthesis method, traces of residual solvents (ppm) can remain in the formulation, despite the most thorough evaporation methods [78]. The effects of residual solvent on GM remain unknown. In addition, DA analysis revealed that many bacterial taxa impacted by *t*FA were also impacted by supplementation with the lecithin vehicle, further hindering our ability to draw conclusions about the effects of *t*FA on GM. Finally, large amounts of variation in group-wise baseline measurements (day 0) of specific taxa may have confounded longitudinal DA results and presented further challenges in interpreting compositional changes to the data. The major strength is the physiological dose of the *t*FA received (2.1–3.2% of total energy intake) which is equivalent to a realistic consumption. However, it is important to note that 76–99% of dietary fat is absorbed in the small intestine and does not reach the large intestine [79]. Therefore, *t*FAs absorbed could further contribute additional effects on health due to other mechanisms.

## 5. Conclusions

To conclude, the impact of *t*FA on GM and fecal metabolites varies depending on the type of *t*FA ingested. Specifically, the findings indicate that the intake of TPA may lead to improvements in the composition of GM and the production of SCFAs, which can confer health benefits to the host. In contrast, EA intake may decrease beneficial microbiomes and SCFAs. However, it is important to note that further research is necessary to fully comprehend the mechanisms underlying these effects and to establish any potential health benefits or biomarkers of health associated with R-*t*FA.

## Data Availability

Supporting data are available from the authors if requested.

## Supplementary Materials

The following supporting information can be downloaded at: https://www.mdpi.com/article/10.3390/nu15061433/s1, Table S1, changes after lecithin, EA, TPA, and water intake after 7 or 28 days. Table S2, Fold-change semi-quantitative metabolites compared to baseline (day 0) in all groups. Figure S1, change in microbiomes after 7 and 28 days. Figure S2, change in metabolites after 7 and 28 days. Figure S3, fold changes for quantitative (Q) metabolites after lecithin, EA, TPA, and water intake after 7 and 28 days. Figure S4, the abundances of all the (consensus) DA taxa across diet groups and timepoints.
