# Supplementary material for: Industrial and Ruminant Trans-Fatty Acids-Enriched Diets Differentially Modulate the Microbiome and Fecal Metabolites in C57BL/6 Mice
Source: Nutrients. 2023 Mar 16;15(6):1433. doi: 10.3390/nu15061433 (PMC10052023; doi:10.3390/nu15061433)
Supplement: Supplementary file 1 [file nutrients-15-01433-s001.zip › nutrients-2259555-supplementary.pdf]

Table S1. Fold-change for quantitative (Q) metabolites compared to day=0 in all groups.

	Comparison within EA group		Comparison within TPA group		Comparison within lecithin group		Comparison within Water group	
	D7	D28	D7	D28	D7	D28	D7	D28
**Carbohydrates**								
Glucose-6-phosphate	-	2.63	2.49	5.05	0.28	6.03	3.49	-
Glucose	75.57	-	-	2.73	-	5.28	-	-
Fructose	-	2.54	2.39	2.34	-	4.36	-	-
Lactose	-	2.81	-	-	-	-	-	-
Maltose	0.38	-	-	3.65	-	7.80	-	-
Mannitol	0.03	3.22	-	-	-	5.41	3.26	2.20
Mannose	25.93	-	2.07	2.50	-	3.97	2.50	-
Sucrose	27.25	-	-	-	-	-	-	-
Sorbitol	0.11	-	-	-	-	-	-	-
**Amino acids**								
Aspartic acid	-	-	-	-		2.07	-	-
Creatinine	-	3.63		0.37	3.15	4.67	-	3.93
Cystathionine	-	-	-	-	-	2.21	-	-
Glutamic acid	-	-	-	2.53	-	2.28	2.37	-
Isoleucine	-	-	-	-	-	2.64	-	-
Leucine	-	-	-	-	-	2.80	-	-
Phenylalanine	-	-	-	-	-	2.27	-	-
3-Aminoisobutyric acid	-	-	-	-	-	2.37	-	-
**Vitamins**				-			-	-
Ascorbic acid	-	-	-	-	-	2.51	2.17	-
Dehydroascorbic acid	-	0.50	-	0.30		-	-	-
Ergocalciferol (Vit D2)	-	-	-	-	2.60	-	-	-
Nicotinic acid (Vit B3)	-	2.51	2.75	3.11	-	3.85	2.97	-
**Other metabolites**	-	-	-				-	-
Citrulline	-	-	-	2.42	-	3.50	-	-
Citric acid	-	-	0.50	-	-	-	-	-
Myo-inositol	-	2.01	-	-	8.14	-	-	3.96
Urea	-		0.16	0.15	-	0.03	-	-
Uric acid	-	-	-	0.36	-	0.29	-	-

Fold changes after Lecithin, EA, TPA, and water intake after 7 days and 28 days. Values below 1 show a decrease in metabolites and more than 1 show an increase in metabolites.

Table S2. Fold-change semi-quantitative metabolites compared to baseline (day 0) in all groups.

	Comparison within EA group		Comparison within TPA group		Comparison within lecithin group		Comparison within Water group	
	D7	D28	D7	D28	D7	D28	D7	D28
**Lipids (SCFA)**								
Propionic acid	-	-	-	2.38	-	-	-	-
Butyric acid	-	-	2.27	4.67	-	-	-	-
Isobutyric acid	-	0.4	-	2.16	0.46	-	-	-
Valeric acid	-	0.4	-	-	0.50	-	-	-
Isovaleric acid	0.45	0.34	-	-	0.41	-	-	-
Isocaproic acid	0.29	0.22	-	-	0.33	0.20	0.40	0.18
Caproic acid		0.38	-	-	-	0.45	-	-
Heptanoic acid	0.40	0.3	-	-	0.42	0.23	0.47	0.27
**Other lipids**								
Azelaic acid	-		-	-	2.09	-	-	-
Elaidic acid	-	-	-	-	-	-	-	2.02
Methylsuccinic acid	-	-	-	-	-	-	-	2.10
Palmitoleic acid	-		-	2.65	2.06	-	2.43	-
Succinic semialdehyde	-	-	-		-	-	2.41	-
9,12-Octadecadienoic acid (Z,Z)-, methyl ester	-	2.02	-	-	-	-	-	-
Myristic acid	-	-	-	-	-	-	-	2.11
**Carbohydrates**								
D-glucose-6-phosphate	0.12	2.51	2.53	5.01	0.29	5.30	3.43	-
Fructose	50.43	2.58	2.39	2.37	-	4.32	2.57	2.08
Glucose	4.81	-	-	2.70	-	5.17	-	-
Lactose	43.73	2.84	-	-	-	2.97	0.39	0.44
Maltose	37.72	-	-	3.53	-	7.43	-	-
Mannitol	0.42	3.43	-	-	-	5.30	3.26	2.21
Mannose	18.71	2.10	2.06	2.36	-	3.69	2.50	-
Ribitol	0.05	-		0.45	-	-		-
Sucrose	24.55	-	-	-	-	-	-	-
Sorbitol	2.49	-	-	-	-	-	-	-
Xylitol	0.05	2.29	-	-	2.18	-	-	0.38
**Amino acids**								
Beta-Glutaric acid	-	-	2.96	-	-	-	-	2.99
Creatinine	-	3.27		0.38	2.91	-	-	3.84
Cystathionine	-	-	2.04	-	-	-	-	3.70
Glutamic acid	-	-	-	2.54	-		2.48	-
Leucine	-	-	-	-	-	2.28	-	-
N-acetyl-D-glucosamine	2.66	-	-	-	-	-	2.02	-
N-acetyl-L-aspartic acid		2.07	-	2.04	-	-		-
N-acetyl-L-glutamic acid	-	-	2.29		-	-	3.31	3.31
Ornithine	-	-	2.28	2.64			-	-
Phosphoserine	-	-	-	-	2.27	-		-
Taurine	5.68	4.15	-	2.17	2.72	-	3.18	-
3-Methylpiperazine-2,5-dione	-	-		2.17	-	-	-	-

**Vitamins**				-			-	-
Ascorbic acid	-	-	-	-	-	2.34	2.26	-
Alpha-Tocopherol acetate	-	-	-			-	-	2.18
Dehydroascorbic acid	-	-	-	0.32		-	-	-
Ergocalciferol (Vit D2)	-	-	-	-	0.15	-	-	-
Nicotinic acid (Vit B3)	-	2.46	2.71	3.08	-	3.69	2.95	-
Pantothenic acid (Vit B5)	-	-		0.28	-	-	-	-
**Other metabolites**	-	-	-				-	-
Alpha ketoglutaric acid	-	-	2.02	-	0.48	3.58	4.12	-
Benzeacetic acid	-	-	-	2.23	2.10	-	2.98	2.01
Bile acid 1	-	-	-	-		-	2.91	-
Bile acid 2	-	-	-	-	3.60	2.94	2.40	-
Bile acid 3	-	-	-	-	3.42	-	-	-
Bile acid 5	-	-	2.44	-	9.04	-	-	-
Bile acid 8	-	-	-	-	2.71	2.27	-	-
Bile acid 9	-	-	-	-	-	2.37	2.64	-
Bile acid 10	-	-	-	-	-	3.46	2.92	-
Citrulline	-	-	-	2.26	-	-	-	-
Flavin adenine dinucleotide	-	-	-	-	2.18	-	-	
Guanosine	-	2.36	3.18	3.06	-	7.75	4.43	2.36
Glycerol 1-phosphate	-	-	2.14		-	3.07	2.33	-
Hydro cinnamic acid	-	-	-	2.49	-	3.16	-	-
Hypotaurine	-	-	6.93	4.16	-	-		-
Hypoxanthine	-	-	3.09	2.67	-	4.44	3.47	-
Inosine	-	2.28	4.24	5.23	0.41	10.57	5.05	2.18
Myo-inositol	-	2.00	0.39	-	8.51	3.39	-	-
Pseudo uridine	-	-	-	-	0.42	2.45	2.66	-
Spermidine	-	-	2.59	-	-	3.66	2.06	-
Uracil	-	-	-	-	-	2.57	2.52	-
Urea	-		0.16	0.15	-	0.03	-	-
Uric acid	-	-	-	0.37	-	0.28	-	-
Uridine	-	3.96	5.30	5.93	-	5.15	5.17	3.96
Urocanic acid	-	-	2.60	-	-	3.42	2.00	2.00
Xanthine	-		-		-	2.60	3.14	-
1-methyl nicotinamide	-	-	-	-	0.37	-	-	-
2-Hydroxyglutaric acid	-	-	-	-	0.37	-	3.79	-
3-Indolepropionic acid	-	-	-	2.51	-	3.24	-	-
3-(3-Hydroxyphenyl) propionic acid	-	-	0.38	-	-	-	0.30	-
3-aminoisobutyric acid	-	-	-	-	-	2.04	-	-
3,4-Dihydroxyphenylacetic acid	-	3.05	-	2.40	0.47	2.91	-	3.05
4-Hydroxybenzeneacetic acid	-	2.61	-	-	0.50	-	-	2.61
5-Hydroxy indole-3-acetic acid	-	-	0.38	-	-	-	0.22	-
6-Hydroxynicotinic acid	-	-	2.57	-	-	6.21	3.77	-

Fold changes after Lecithin, EA, TPA, and water intake after 7 or 28 days. Values below 1 show a decrease in metabolites and more than 1 show an increase in metabolites. **SCFA:** short-chain fatty acid, change in all values are significant compare to day 0 ($P<0.5$).

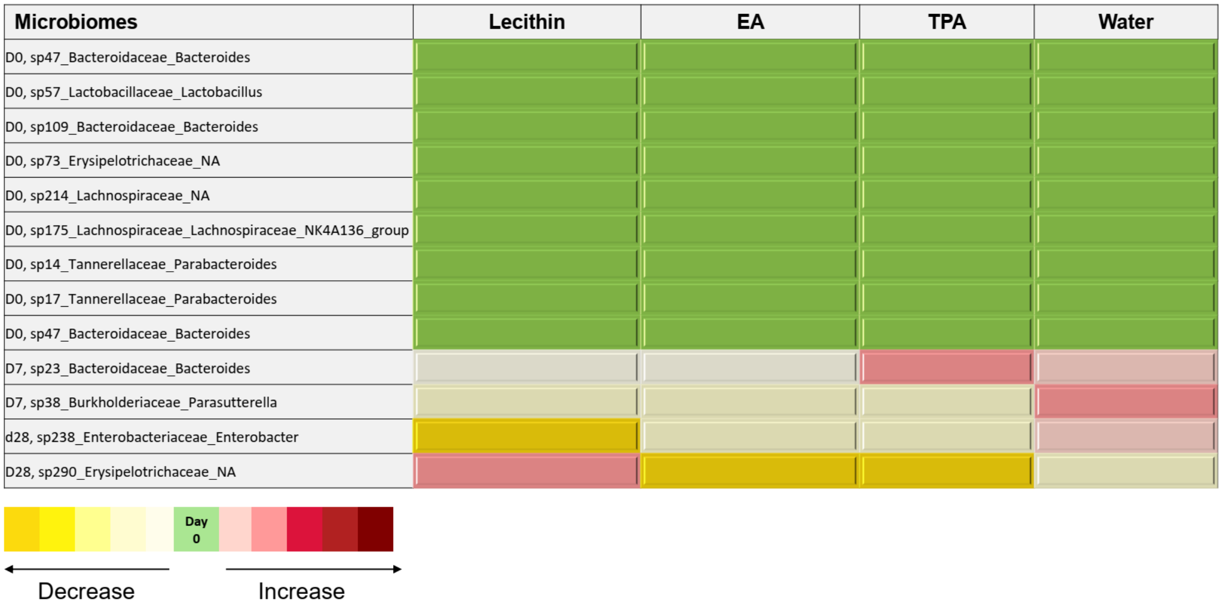

Figure S1. Change in microbiomes after 7 and 28 days, data from machine learning

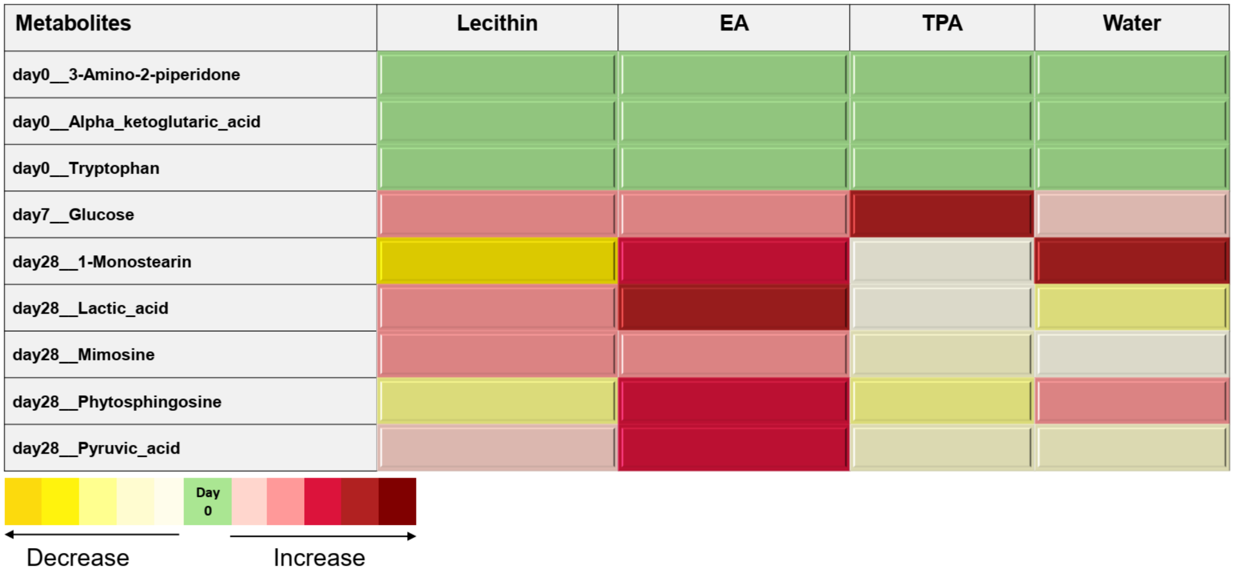

Figure S2. Change in metabolites after 7 and 28 days, data from machine learning

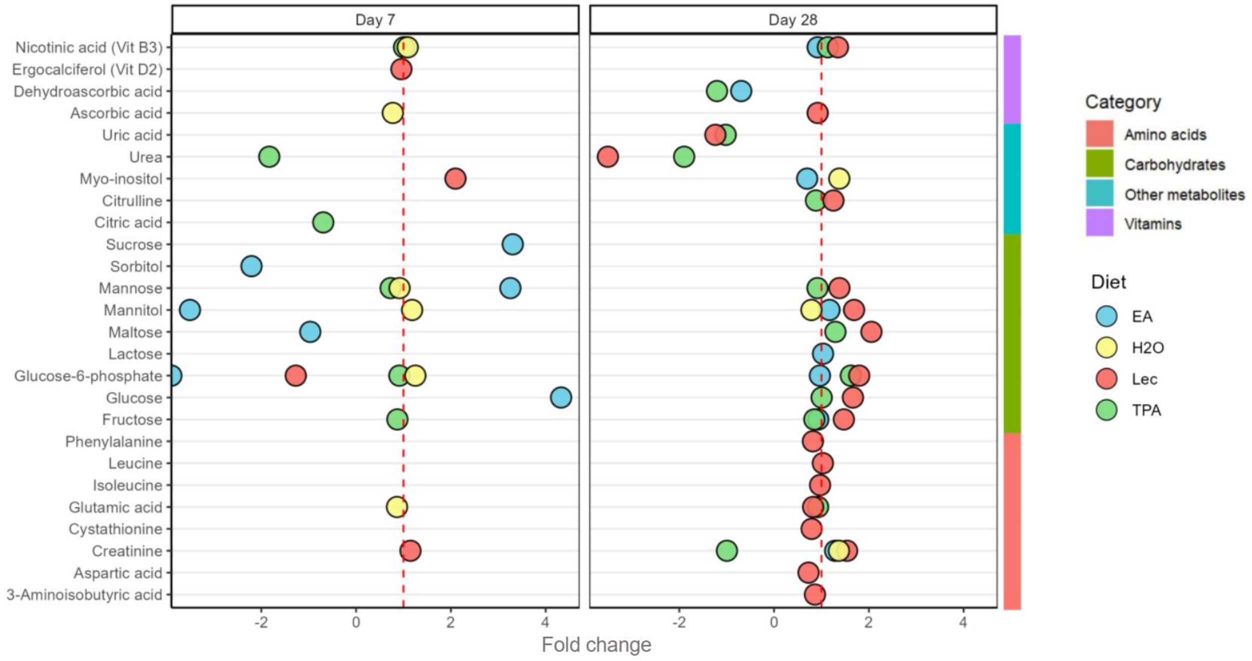

Figure S3. Fold changes Fold-change for quantitative (Q) metabolites after lecithin, EA, TPA, and water intake after 7 days and 28 days. Values below 1 show a decrease in metabolites and more than 1 show an increase in metabolites.

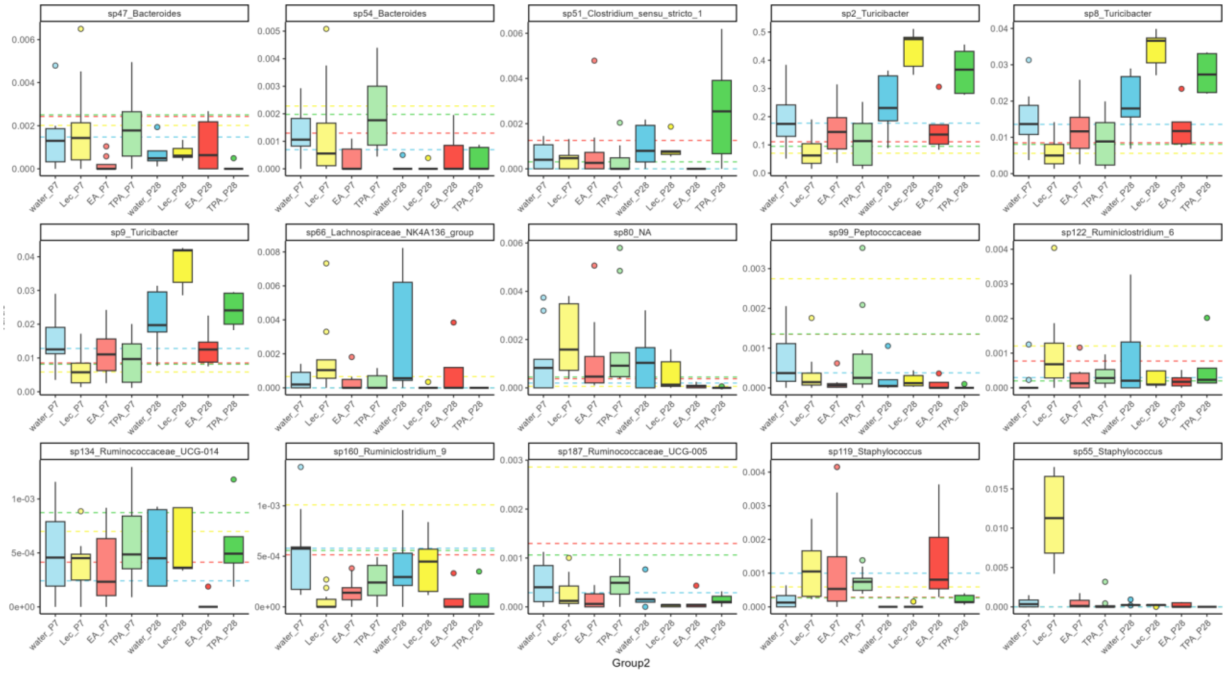

Figure S4. The abundance of all the (consensus) DA taxa across diet groups and timepoints.
